# Impact of soda tax on beverage price, sale, purchase, and consumption in the US: a systematic review and meta-analysis of natural experiments

**DOI:** 10.3389/fpubh.2023.1126569

**Published:** 2023-09-22

**Authors:** Jing Shen, Junjie Wang, Fan Yang, Ruopeng An

**Affiliations:** ^1^Department of Physical Education, China University of Geosciences (Beijing), Beijing, China; ^2^School of Kinesiology and Health Promotion, Dalian University of Technology, Dalian, Liaoning, China; ^3^School of Public Administration, Dongbei University of Finance and Economics, Dalian, Liaoning, China; ^4^Brown School, Washington University, St. Louis, MO, United States

**Keywords:** sugar-sweetened beverages, tax, systematic review, meta-analysis, natural experiments

## Abstract

**Background:**

As a primary source of added sugars in the US diet, sugar-sweetened beverage (SSB) consumption is presumed to contribute to obesity prevalence and poor oral health. We systematically synthesized and quantified evidence from US-based natural experiments concerning the impact of SSB taxes on beverage prices, sales, purchases, and consumption.

**Methods:**

A keyword and reference search was performed in PubMed, Web of Science, Cochrane Library, Scopus, and EconLit from the inception of an electronic bibliographic database to Oct 31, 2022. Meta-analysis was conducted to estimate the pooled effect of soda taxes on SSB consumption, prices, passthrough rate, and purchases.

**Results:**

Twenty-six natural experiments, all adopting a difference-in-differences approach, were included. Studies assessed soda taxes in Berkeley, Oakland, and San Francisco in California, Philadelphia in Pennsylvania, Boulder in Colorado, Seattle in Washington, and Cook County in Illinois. Tax rates ranged from 1 to 2 ¢/oz. The imposition of the soda tax was associated with a 1.06 ¢/oz. (95% confidence interval [CI] = 0.90, 1.22) increase in SSB prices and a 27.3% (95% CI = 19.3, 35.4%) decrease in SSB purchases. The soda tax passthrough rate was 79.7% (95% CI = 65.8, 93.6%). A 1 ¢/oz. increase in soda tax rate was associated with increased prices of SSBs by 0.84 ¢/oz (95% CI = 0.33, 1.35).

**Conclusion:**

Soda taxes could be effective policy leverage to nudge people toward purchasing and consuming fewer SSBs. Future research should examine evidence-based classifications of SSBs, targeted use of revenues generated by taxes to reduce health and income disparities, and the feasibility of redesigning the soda tax to improve efficiency.

## 1. Introduction

Sugar-sweetened beverages (SSBs) are beverages sweetened with various forms of added sugars, including brown sugar, corn syrup, glucose, lactose, and sucrose (1). In the United States, SSB consumption is prevalent, with six in ten youths and five in ten adults consuming SSBs on any given day during the period of 2011–2014 (2, 3). This consumption amounts to over 140 kcal from SSBs per day for both youth and adults (2, 3). Extensive epidemiologic studies have consistently documented that SSB consumption, as a primary source of added sugars in the US diet, is a significant contributor to the prevalence of obesity, cardiometabolic diseases, and oral health risk (4–6). These associations have also been observed globally (7, 8).

Recent policy interventions aimed at combating obesity have utilized economic incentives to “nudge” (i.e., promote or encourage) people toward a healthier diet choice (9–11). For example, healthy food subsidies promote fruit and vegetable intake, while excise taxes aim to discourage the consumption of less desirable foods and beverages. Excise taxes, such as soda taxes, are implemented in countries like the US, France, New Zealand, Netherlands, and South Africa (12). A soda tax is a specific excise tax charged on the sale of SSBs to reduce consumption (13). Merchants pay the tax, which is then passed on to consumers through higher prices. The amount of the tax varies across different regions, as it is applied by both state and federal governments.

Preliminary evidence suggests that soda taxes are associated with weight loss, reduced body mass index (BMI), and decreased risks of overweight and obesity (14, 15). Additionally, soda taxes can help address dental health issues and reduce the prevalence of tooth decay (16). Berkeley, California, became the first city in the US to implement a soda tax, which imposed a 1 ¢/oz. tax on the distributors of specific SSBs, including soda and sports/energy drinks (17). This tax took effective on January 1, 2015 (17). Following Berkeley’s example, several other cities, such as Philadelphia, Pennsylvania (1.5 ¢/oz.; 1/1/2017), Albany, California (1 ¢/oz.; 4/1/2017), Oakland, California (1 ¢/oz.; 7/1/2017), Boulder, Colorado (2 ¢/oz.; 7/1/2017), Cook County, Illinois (1 ¢/oz.; 8/2/2017), San Francisco, California (1 ¢/oz.; 1/1/2018), and Seattle, Washington (1.75 ¢/oz.; 1/1/2018), have also implemented soda taxes (18).

Existing research on the effect of soda taxes in the US can be categorized into three groups: “proxy,” “modeling,” and “local” studies. The “proxy” studies have used state sales tax on soda, candy, and other qualified groceries as a substitute for a soda tax due to the absence of soda taxes or related data (19–24). However, using such a “proxy” can be problematic because a soda tax, as an excise tax, is fundamentally different from a state general or selective sales tax applied as a percentage of the purchase price (24). The “modeling” studies have employed systems science models to simulate the effect of a soda tax using pre-specified parameter values and statistical distributions (25–34). However, these studies differ from other categories of research in that they are prospective in nature. They aim to identify potential effects of policies that have yet to be enacted and, by necessity, make assumptions about possible retailer and consumer responses. Although the “modeling” studies compare simulated counterfactuals to status quo baselines, they do not provide direct causal inference due to their non-experimental study design. The “local” studies, on the other hand, have utilized quasi-experimental methods to compared soda prices, sales, purchases, or consumption between cities that have implemented a soda tax and neighboring cities without such a tax, or before and after the implementation of a soda tax in a city. Unlike “proxy” or “modeling” studies, the “local” studies directly estimated the impact of soda tax using temporal and geographical variations in tax implementation (35–52). Thus, these “local” studies serve as natural experiments that provide valuable causal inference. Powell et al. conducted a review of seven local SSB tax implementations and reported an average tax pass-through rate of 70% (53). Another review by Powell et al. found that the demand for SSBs declined by 20%, with an estimated price elasticity of demand of −1.5 following the implementation of soda taxes (54).

This study aims to systematically synthesize and quantify the impacts of soda taxes on beverage prices, sales, purchases, and consumption in the US. By exclusively relying on natural experiments (i.e., “local” studies), our review provides robust causal inferences regarding the effectiveness of soda taxes. Moreover, our approach goes beyond a narrative review by providing quantitative estimates of the magnitude of the tax effect. The findings of this study can inform local, state, and federal policymakers in designing or revising soda tax-related legislation and implementation strategies to effectively combat obesity.

## 2. Methods

The present study was conducted following the Preferred Reporting Items for Systematic Reviews and Meta-Analyses (55).

### 2.1. Eligibility criteria

Studies meeting all of the following criteria were eligible for the review: (1) Participants: consumers, stores, or beverage items within US taxing jurisdictions that implemented a soda tax; (2) Interventions: soda tax (SSB excise tax); (3) Comparisons: consumers, stores, or beverage items within and outside the US taxing jurisdictions that implemented a soda tax; (4) Outcomes: beverage prices, sales, purchases, and consumption; (5) Study design: natural experiment; (6) Article type: peer-reviewed original study; (7) Time window of search: from the inception of an electronic bibliographic database to Oct 31, 2022; and (8) Language: English.

### 2.2. Search strategy

A keyword search was performed in five electronic bibliographic databases: PubMed, Web of Science, Cochrane Library, Scopus, and EconLit. The search algorithm included all keywords from two groups: (1) “tax,” “taxes,” “taxation,” “taxed,” “taxing,” “pre-taxation,” “post-taxation,” “pre-tax,” “post-tax,” “excise,” or “excises”; and (2) “beverage,” “beverages,” “drink,” “drinks,” “soda,” “sodas,” “cola,” “coke,” “SSB,” or “SSBs.” The search algorithm used in PubMed was reported in Appendix 1 (Supplementary material). Two co-authors independently screened the title and abstract and identified potentially pertinent articles for the full-text review (Cohen’s kappa κ = 0.85).

### 2.3. Meta-analysis

Meta-analyses were performed to estimate the pooled effects, represented as mean differences, of soda taxes in the US. The six outcomes included: (1) change in prices of taxed beverages (i.e., SSBs); (2) change in prices of untaxed beverages; (3) change in purchases of taxed beverages; (4) change in purchases of untaxed beverages; (5) change in the consumption of taxed beverages; and (6) tax passthrough rate. Out of the 40 studies, 13 were excluded from the meta-analyses due to: (1) non-overlapping outcome measures (36, 40, 44, 56–63), or (2) neither standard error nor confidence interval (CI) reported (35, 43). To assess heterogeneity among the included studies, we employed the *I*^2^ index, which allows us to quantify the degree of variability between study estimates (64). The *I*^2^ index was interpreted as modest (*I*^2^ ≤ 25%), moderate (25% < *I*^2^ ≤ 50%), substantial (50% < *I*^2^ ≤ 75%), or considerable (*I*^2^ > 75%) (65). Based on the level of heterogeneity observed, we estimated the meta-analyses using either a fixed-effect (FE) model or a random-effect (RE) model. The FE model was utilized when modest or moderate heterogeneity was present, while the RE model was employed when substantial or considerable heterogeneity was observed. To assess publication bias, we conducted Begg’s and Egger’s tests. These tests allow us to evaluate potential bias in the included studies (66). Additionally, we performed random-effect meta-regressions to assess the dose–response effect of alternative soda tax rates on the various outcomes. Meta-analyses were performed using Stata 16.1 MP version. We employed these methods to synthesize the available evidence and provide quantitative estimates of the pooled effects of soda taxes on the specified outcomes.

### 2.4. Study quality assessment

Following Littell et al. (65) and An et al. (67), we designed a study quality assessment tool that rated each study based on ten criteria (Table 5). For each criterion, a score of 1 is assigned if the answer is “yes”; otherwise, a score of 0 is assigned if the answer is “no,” “not applicable” or “not reported.” We sum the scores of all ten criteria for a total study-specific score, ranging from 0 to 10. The study quality score was not used as a criterion for inclusion of a study, but as a measure of the strength of the scientific evidence.

## 3. Results

### 3.1. Study selection

**Figure 1:**
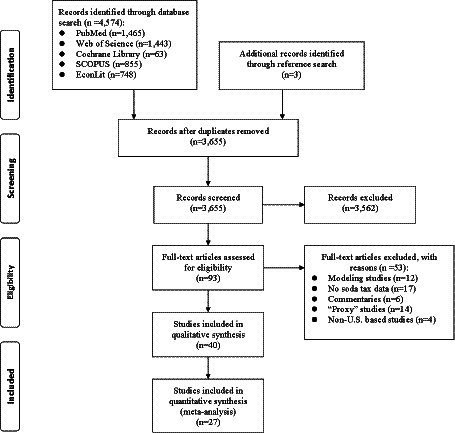
Study selection flow diagram (PRISMA).

Figure 1 shows the study selection flow diagram. We identified 4,574 articles through the keyword and reference search. After removing duplicates, 3,655 articles underwent title and abstract screening, in which 3,562 articles were excluded. The remaining 93 articles were reviewed of full texts against the eligibility criteria. Of these, 53 articles were excluded, including 12 “modeling” studies, 14 “proxy” studies, 17 studies that did not examine soda taxes, six commentaries, and four non-US-based studies. The remaining 40 studies that examined the impact of soda taxes on beverage prices, sales, purchases, and consumption in the US were included in the review (35–52, 56–63, 68–81).

### 3.2. Characteristics of the included studies

Table 1 reports the characteristics of soda taxes assessed in the included studies. All 40 studies were conducted in or after 2015. Fourteen examined soda tax in Philadelphia, Pennsylvania (35, 39, 40, 43, 49, 50, 52, 57, 58, 60, 68, 69, 76, 81); eight in Berkeley, California (36, 37, 44–47, 51, 56); six in Oakland, California (42, 61, 72, 77, 78, 80); three each in Cook County, Illinois (73–75), and Seattle, Washington (48, 71, 79); two in Boulder, Colorado (38, 59); one each in two cities—Oakland and San Francisco (70), Berkeley and Washington State (62); one in six cities—Philadelphia, Berkeley, Seattle, Boulder, Cook County, and Oakland (63); and the remaining one in four cities—Philadelphia, San Francisco, Seattle, and Oakland (41). The tax rate ranged from 1 to 2 ¢/oz., with a mean of 1.321 ¢/oz. among the seven cities under investigation.

**Table 1:** Characteristics of soda taxes assessed in the studies included in the review.

Study ID	Author, Year	Taxing jurisdiction	Implementation date	Tax rate	Assessment Period
1	Falbe et al. (45)	Berkeley, CA	Jan 1, 2015	1 ¢/oz	Pre-tax: Nov 2014 through Jan 2015 Post-tax: May through Jun 2015
2	Falbe et al. (46)	Berkeley, CA	Jan 1, 2015	1 ¢/oz	Pre-tax: Apr through Jul 2014 Post-tax: Apr through Aug 2015
3	Cawley and Frisvold (37)	Berkeley, CA	Jan 1, 2015	1 ¢/oz	Pre-tax: Dec 22, 2014 Post-tax: Jun 1, 2015
4	Debnam (44)	Berkeley, CA	Jan 1, 2015	1 ¢/oz	2010 through 2015
5	Silver et al. (51)	Berkeley, CA	Jan 1, 2015	1 ¢/oz	1. Prices data: Pre-tax: Dec 2014 Post-tax: Jun 2015 and Mar 2016 2. Point of electronic sale data: Jan 1, 2013, through Feb 29, 2016
6	Bollinger and Sexton (36)	Berkeley, CA	Jan 1, 2015	1 ¢/oz	Jan 2013 through Dec 2015
7	Cawley et al. (68)	Philadelphia, PA	Jan 1, 2017	1.5 ¢/oz	Pre-tax: December 21, 2016 Post-tax: January 14, 2017, and February 5, 2017
8	Cawley et al. (68)	Boulder, CO	Jan 1, 2017	2 ¢/oz	Pre-tax: Apr through Jun 2017 Post-tax: Aug through Oct 2017
9	Coary and Baskin (43)	Philadelphia, PA	Jan 1, 2017	1.5 ¢/oz	Pre-tax: Dec 2016 Post-tax: Sep 2017
10	Zhong et al. (52)	Philadelphia, PA	Jan 1, 2017	1.5 ¢/oz	Pre-tax: Dec 6 through Dec 31 2016 Post-tax: Jan 15 through Feb 31, 2017
11	Baskin and Coary (35)	Philadelphia, PA	Jan 1, 2017	1.5 ¢/oz	Pre-tax: Nov 2015 through Feb 2016 Post-tax: Nov 2016 through Feb 2017
12	Cawley et al. (41)	1. Philadelphia, PA 2. San Francisco, CA 3. Seattle, WA 4. Oakland, CA	1. Jan 1, 2017 2. Jan 1, 2018 3. Jan 1, 2018 4. Jul 1, 2017	1. 1.5 ¢/oz. 2. 1 ¢/oz. 3. 1.75 ¢/oz. 4. 1 ¢/oz	Jul 1, 2016 through Jun 30, 2017
13	Cawley et al. (40)	Philadelphia, PA	Jan 1, 2017	1.5 ¢/oz	Pre-tax: Nov through Dec 2016 Post-tax: Nov through Dec 2017
14	Lee et al. (47)	Berkeley, CA	Jan 1, 2015	1 ¢/oz	Pre-tax: Apr through Jul 2014 Post-tax: Apr through Oct 2017
15	Roberto et al. (49)	Philadelphia, PA	Jan 1, 2017	1.5 ¢/oz	Jan 1, 2014 through Dec 31, 2017
16	Taylor et al. (56)	Berkeley, CA	March 1, 2015 (not on campus) August 2016 (on campus)	1 ¢/oz	The pre-election campaign period: July 2014 through October 2014 The postelection and pretax implementation period: November 2014 through February 2015 The tax implementation period not on campus: March 2015 through July 2016 The tax implementation period on campus: August 2016 through December 2016
17	Bleich et al. (69)	Philadelphia, PA	Jan 1, 2017	1.5 ¢/oz	Pre-tax: Oct through Dec 2016 Post-tax: Jun through Aug 2017, Oct through Dec 2017
18	Cawley et al. (40)	Oakland, CA	Jul 1, 2017	1 ¢/oz	Pre-tax: Apr through Jun 2017 Post-tax: Apr through Jun 2018
19	Cawley et al. (40)	Philadelphia, PA	Jan 1, 2017	1.5 ¢/oz	Pre-tax: Nov through Dec 2016 Post-tax: Nov through Dec 2017
20	Falbe et al. (70)	Oakland, CA San Francisco, CA	Jul 1, 2017 Jan 1, 2018	1 ¢/oz	Pre-tax: Apr through May 2017 Post-tax: Apr through May 2018
21	Jones-Smith et al. (71)	Seattle, WA	Jan 1, 2018	1.75 ¢/oz	Pre-tax: Oct through Nov 2017 Post-tax: May through Jul 2018
22	Lawman (57)	Philadelphia, PA	Jan 1, 2017	1.5 ¢/oz	Pre-tax: Sept through Dec 2016 Post-tax: 3, 6, and 12 months after tax implementation
23	Marinello et al. (78)	Oakland, CA	Jul 1, 2017	1 ¢/oz	Pre-tax: May through Jun 2017 Post-tax: Jan 2018 and Jun 2018
24	Powell and Leider (73)	Cook County, IL	August 2, 2017	1 ¢/oz	Pre-tax: March 29, 2015 through July 29, 2017 Post-tax: August 6, 2017 through November 25, 2017 Post-repeal: December 3, 2017 through August 4, 2018
25	Powell et al. (74)	Cook County, IL	Aug 2, 2017	1 ¢/oz	Pre-tax: Aug 7, 2016, to Nov 26, 2016 Post-tax: Aug 6, 2017, to Nov 25, 2017
26	Powell and Leider (48)	Seattle, WA	Jan 1, 2018	1.75 ¢/oz	Pre-tax: Sep 29, 2017 through Feb 4, 2018 Post-tax: Feb 4, 2018 through Sep 29, 2018
27	Powell et al. (75)	Cook County, IL	Aug 2, 2017	1 ¢/oz	Pre-tax: Aug 7, 2016, to Nov 26, 2016 Post-tax: Aug 6, 2017, to Nov 25, 2017
28	Zhong et al. (58)	Philadelphia, PA	Jan 1, 2017	1.5 ¢/oz	Pre-tax: Dec 2016 through Jan 2017 Post-tax: Dec 2017 through Feb 2018
29	Bleich et al. (58)	Philadelphia, PA	Jan 1, 2017	1.5 ¢/oz	Baseline: October through December 2016 Post-tax: 6, 12, and 24 months after tax implementation
30	Cawley et al. (59)	Boulder, CO	July 1, 2017	2 ¢/oz	Pre-tax: April and June 2017 Post-tax: August and October 2017
31	Edmondson et al. (60)	Philadelphia, PA	Jan 1, 2017	1.5 ¢/oz	September 2012 through December 2019
32	Léger and Powell (77)	Oakland, CA	Jul 1, 2017	1 ¢/oz	Pre-tax: July 1, 2016 to June 30, 2017 Post-tax: July 1, 2017 to June 30, 2018
33	Léger and Powell (77)	Oakland, CA	Jul 1, 2017	1 ¢/oz	Pre-tax: late May–June 2017 Post-tax: June 2019
34	Marinello et al. (78)	Oakland, CA	Jul 1, 2017	1 ¢/oz	Pre-tax: May through June 2017 Post-tax: June 2019
35	Powell et al. (53, 54)	Seattle, WA	Jan 1, 2018	1.75 ¢/oz	Pre-tax: 2017 Post-tax: February 3, 2019 through September 28, 2019
36	Rojas and Wang (62)	1. Berkeley, CA 2. Washington State	1. March 1, 2015 2. July 1, 2010	1 ¢/oz. 0.166¢/oz	1. January 2014 through December 2015 2. January 2009 through December 2012
37	Seiler et al. (50)	Philadelphia, PA	Jan 1, 2017	1.5 ¢/oz	Jan 2015 through Sep 2018
38	Zhang et al. (63)	1. Seattle, WA 2. Boulder, CO 3. Cook County, IL 4. Philadelphia, PA 5. Berkeley, CA 6. Oakland, CA	1. Jan 2018 2. Jul 2017 3. Aug 2017 4. Jan 2017 5. Mar 2015 6. Jul 2017	1. 1.75 ¢/oz. 2. 2 ¢/oz. 3. 1 ¢/oz. 4. 1.5 ¢/oz. 5. 1 ¢/oz. 6. 1 ¢/oz	From 2013 to 2018
39	Leider and Powell (80)	Oakland, CA	Jul 1, 2017	1 ¢/oz	Pre-tax: July 31, 2016 through May 27, 2017 Post-tax: July 29, 2018 through May 25, 2019
40	Petimar et al. (81)	Philadelphia, PA	Jan 1, 2017	1.5 ¢/oz	From January 1, 2016 to December 30, 2018

To account for cross-border estimation across studies within the same jurisdiction, we employed a variety of methods to explain the variation in findings. These methods included considering differences in sample size, sample characteristics, statistical approaches, and outcome measures. By examining these factors, we aimed to provide insights into the varying effects observed within jurisdictions. Table 2 reports sample characteristics, statistical approach, and outcome measures of the studies included in the review. Twenty-nine studies collected beverage pricing or sales data from various types of retailers (e.g., supermarkets, corner/convenience stores, restaurants, and grocery stores) (35–39, 43, 45, 48–51, 56, 59, 61–63, 68–75, 77–81), whereas the remaining 11 surveyed participants regarding their beverage purchases or consumption (40–42, 44, 46, 47, 52, 57, 58, 60, 76). All studies except one (73) adopted a difference-in-differences (DID) approach to estimate the impacts of soda taxes. DID is a quasi-experimental approach (82), which uses geographical and timing variations in soda tax implementation across US cities to estimate the causal impact of soda taxes on SSB prices, sales, purchases, and consumption. Twenty-eight studies assessed the effect of soda taxes on SSB or untaxed beverage prices (36–39, 42, 43, 45, 48–51, 59, 61–63, 68–73, 75–81). Twenty-two studies focused on beverage sales or purchases (35–37, 41, 42, 44, 48–51, 56, 57, 62, 63, 69, 73, 74, 76, 77, 79–81). Nine studies examined the frequency or quantity of beverage consumption (40, 42, 44, 46, 47, 51, 52, 58, 60). Data on beverage prices were collected mainly through three channels: hand-recoding of price tags during retailer visits (37–39, 42, 43, 45, 59, 61, 68–72, 76, 78), web-scraped data of beverage prices (38, 59), or point-of-sale electronic scanner data (36, 48–51, 59, 62, 63, 73, 75, 77, 79–81). Data on beverage sales were collected through two channels: retailers’ aggregate sales records (35, 42, 56) or point-of-sale electronic scanner data (36, 48–51, 62, 63, 73, 74, 77, 79–81). Data on beverage purchases were collected from surveyed participants (40, 41, 44, 57, 69, 76). Data on beverage consumption were collected through two methods: interviews using a food frequency questionnaire (40, 46, 60) or a 24-h dietary recall (42, 51, 58).

**Table 2:** Sample characteristics, sample size, and measures of the studies included in the review.

Study ID	Sample size	Sample characteristics	Statistical approach	Outcomes assessed	Outcome measures
1	71 retailers	Chain supermarket, small grocery store, drugstore, convenience store, liquor store	DID	Changes in prices of SSBs and non-SSBs	1. Trained research assistants collected beverage prices by recording visible prices from the price tag 2. For beverages without visible prices, data collectors asked store clerks for prices 3. If clerks were uncooperative, data collectors purchased beverages and recorded prices from receipts 4. If a temporary promotional price was advertised, data collectors recorded both the promotional and regular price
2	2,679 participants	Low-income and minority population	DID	Changes in beverage consumption	Beverage consumption via interviewer-administered intercept surveys with a beverage frequency questionnaire modified from the Behavioral Risk Factor Surveillance System 2011 SSB module
3	86 stores	Supermarkets, grocery stores, pharmacies, convenience stores, and gas stations with posted prices	DID	Price of SSBs	Store visits
4	2,399,897 household-purchase-weeks	Households	DID (fixed-effect regression)	Household purchases	1. Data from the Nielsen Consumer Panel 2. For purchases made at retailers where Nielsen does not receive the point of sale data, the panelist manually enters the expenditure made on the purchase
5	1. 26 stores 2. 2,175 store prices 3. Sales data covered 118.8 million barcode scans from 15.5 million transactions	1. Large supermarkets, small chain supermarkets, chain and independent gas stations, pharmacies, and independent corner stores 2. Store price collected for a standard panel of 70 beverages, which included 45 taxed and untaxed branded beverages 3. Point-of-sale electronic scanner data	DID (fixed-effect regression)	Changes in prices, beverage sales, and usual beverage intake	1. Data collection protocols were employed to measure beverage prices systematically 2. Point-of-sale electronic scanner data were requested using personal outreach 3. Trained interviewers used standardized questionnaires and computer-assisted telephone interviews to conduct a 24-h recall of beverage intake 4. A second 24-h beverage recall interview was collected 3–7 days later
6	1. 3,549 UPCs 2. 196,226 weekly UPC prices and sale quantities	UPCs in retail chains	DID	Weekly average prices, sales quantities and UPC (product) volume	Nielsen scanner data
7	31 stores	Retail chains (including bakeries, restaurants, and newsstands)	DID	Beverage prices	Data collected in person at retail stores
8	1. Retailers: 1,035 2. Restaurants: 1,263 3. OrderUp: 158	All retail stores, all limited-service restaurants, and a selected sample of restaurant menus	DID	The changes in beverage prices	1. Hand-collected data of listed prices and purchase prices of beverages from all retail stores 2. Hand-collected data of listed prices of fountain drinks and coffee drinks from all limited-service restaurants 3. Web-scraped data of prices from a selected sample of restaurant menus
9	249 products: 190 soda products, 38 juice products, and 21 water products	Soda, juice, and water	DID	Beverage prices	Data collected from 2 matched stores from each category both inside and outside Philadelphia county
10	1,777 participants	Residents	DID	The daily quantity of consumption, and 30-day average consumption frequency and quantity	Phone-based survey: Beverage questions were based on a modified version of the 15-item Beverage Intake Questionnaire
11	9 stores	Predominant grocery retailers (top 3 in total retail sales)	DID	Total beverage sales by category	Data came from the retailers
12	1,447 households	Households with children	DID	Households’ monthly beverage purchases	Data collected by InfoScout
13	1. Purchase: 1,305 individuals in 2016 and 1,501 in 2017 2. Household survey: 440	Consumers at stores, households with children, and residents	DID	1. Changes in the volume of taxed and untaxed beverages purchased 2. Beverage consumption	1. Interviewed consumers at stores 2. Household survey of beverage consumption based on telephone and online using the National Health and Nutrition Examination Survey Dietary Screener Questionnaire
14	5,225 participants	Residents	DID	SSB and water consumption	SSB consumption was measured annually through beverage frequency questionnaires. The BFQs were based on the previously validated BEVQ-15
15	291 stores	Chain retailers: supermarket, mass merchandizers, and pharmacies	DID	Change in taxed beverage prices and volume sales	Data were purchased from IRI
16		University retailers	DID	The change in soda sales	Panel data of beverage sales from university retailers
17	134 stores and 4,584 purchases	Independent stores	DID	Beverage prices and purchases	Research staff documented beverage prices and collected purchase data from consumers
18	126 stores	Stand-alone convenience stores, gas stations with convenience stores, small and large grocery stores, and pharmacies	DID	Beverage prices, purchases, and consumption	Data collected from stores
19	1. 3,152 prices for 2016 2. 2,763 prices for 2017	Prices from both taxed beverage and untaxed beverage	DID	Changes in beverage prices	Data collected in person at retail stores
20	155 stores	Supermarkets, drugstores, mass merchandizers, convenience stores, corner and small grocery stores, and liquor stores	DID (fixed-effect regression)	Retail prices of SSBs and non-SSBs	Data collectors recorded beverage shelf prices in stores
21	407 stores	Retail food stores, quick-service restaurants, and coffee shops	DID	Beverage prices	Data collected from stores and restaurants
22	603 participants	Adult SSB consumers	DID (mixed-effect regression)	Beverage purchases	Receipts collected from consumers
23	150 observations of bottled regular soda from 39 restaurants, 106 observations of bottled diet soda from 32 restaurants, and 501 observations of fountain drinks from 73 restaurants	Fast-food restaurants	DID	Beverage prices	Data were collected in-person using the Beverage Tax Fast-food Restaurant Observation Form
24	16, 510 UPCs for volume and 2,141 UPCs for price	Taxed and untaxed UPCs	Interrupted time series	Changes in beverage prices and volume sold	Nielsen food store scanner data
25	7,798 UPCs	Taxed and untaxed UPCs within the sites and in the 2-mile border area of each site	DID	The volume sold of taxed and untaxed beverages	Retail scanner data on sales of non-alcoholic beverages were obtained from the Nielsen panel survey
26	7,868 UPCs	Taxed and untaxed UPCs within the sites and in their 2-mile border areas	DID	Beverage prices and volume sold	UPC-level retail store scanner data obtained from the Nielsen
27	13,015 UPCs	Distinct beverage UPCs sold	DID	Beverage prices	Retail scanner data on sales of non-alcoholic beverages were obtained from the Nielsen panel survey
28	515 participants	Residents aged 18–64 years	DID	Change in beverage consumption	A random-digit-dialing phone survey was administered to a population-based cohort
29	116 independent stores and 4,738 customer purchases	Independent stores	DID	Changes in the prices and purchases of beverages	Data were collected by research assistants
30	621 retailers 36 stores 776 restaurants 219 restaurants	Retailers, liquor stores, restaurants	DID	Pass-through of sugar sweetened beverages taxes	Data were collected from four sources: (a) handcollected data on prices from stores; (b) Nielsen Retail Scanner Data of store prices; (c) hand-collected data on prices in restaurants; and (d) web-scraped data from online restaurant menus.
31	86,928 participants	High School Students	DID	Soda consumption	Data were obtained from Youth Risk Behavior Surveillance System
32	2,187 UPCs	Taxed and untaxed UPCs in supermarkets, grocery stores, convenience stores, drug stores, mass merchandize stores, and dollar stores	DID	Changes in beverage prices and volume sold	Data were obtained from custom-ordered Nielsen retail scanner data
33	210 stores	Seven store types: general merchandize stores, supermarkets, grocery stores, chain convenience stores, non-chain convenience stores, small discount stores, and drug stores/pharmacies	DID	Change in beverage prices	Data were collected from stores by the data collector
34	85 restaurants	Chain and non-chain fast-food restaurants	DID	Change in prices of beverages sold	Data were collected at restaurants
35	6,652 UPCs	Taxed and untaxed UPCs in Grocery, drug, convenience, dollar, and mass merchandize stores, and supermarkets	DID	Changes in beverage prices and volume sold	Data were obtained from Nielsen
36	1,503 stores	Grocery stores, drug stores, mass merchandizers, etc.	DID	Changes in beverage prices and volume sold	Data were obtained from the Nielsen retail scanner database
37	357 stores	Chain stores	DID	Beverage prices and quantities sold	Retail scanner data collected by IRI
38	3,719 stores	Supermarkets and convenient stores	DID	Changes in beverage prices and sales quantity	Data from IRI, the InfoScan, store-based scanner data
39	11,705 UPCs	Distinct beverage UPCs sold	DID	Changes in beverage prices and volume sold	Data were obtained from Nielsen
40	109 supermarkets, 45 mass merchandizers, and 350 pharmacies	Large retailers	DID	Changes in beverage price and sales	Data from IRI

IRI, Information Resources, Inc.; DID, difference-in-differences; SSB, sugar-sweetened beverage.

### 3.3. Study findings

Table 3 reports the estimated effects of soda taxes. Four key findings have emerged. First, soda taxes led to increased SSB prices in the taxing jurisdictions relative to the nontaxing jurisdictions. In Berkeley, a 1.0 ¢/oz. soda tax increased the price for taxed beverages by 0.65¢/oz. relative to non-Berkeley stores (51). Across all brands and sizes of SSBs, the overall price of taxed beverages increased by 0.43 ¢/oz. relative to neighboring cities (37). The price increase was 0.47 ¢/oz. for small-size SSBs (≤33.8 oz), 0.46 ¢/oz. for a 2-liter SSB bottle, and 0.49 ¢/oz. for a multipack of soda relative to that in neighboring cities (45). In other taxing jurisdictions implementing a 1.0 ¢/oz. soda tax, Falbe et al. documented that the average price of SSBs increased by 0.92 ¢/oz. (95%CI = 0.28, 1.56; *p* < 0.01) in Oakland 10 months post-tax and 1 ¢/oz. (95%CI = 0.35, 1.65; p < 0.01) in San Francisco 4 months post-tax in comparison to Richmond and San Jose (70). Studies reported that the taxed beverage prices increased by 0.49 ¢/oz. (77), and the regular soda price increased by 0.82 ¢/oz. (72) in Oakland relative to Sacramento one year post-tax. The taxed beverage prices increased by 0.67 ¢/oz. (80), and bottled regular soda prices increased by 1.44 ¢/oz. (78) in Oakland relative to Sacramento two year post-tax. Powell and Leider found that the taxed beverage prices increased by 1.13 ¢/oz. in Cook County relative to St. Louis (73). Powell et al. found the prices of all taxed beverage types increased by 1.19 ¢/oz. in Cook County relative to St. Louis (75). In Philadelphia, a 1.5 ¢/oz. soda tax raised prices by 1.58 ¢/oz. for all taxed beverages combined relative to comparison untaxed stores outside Philadelphia (39). Coary et al. found that SSB prices increased by 1.53 ¢/oz. relative to those outside the city (43). Seiler et al. reported that the soda tax passed through at a rate of 97%, leading to a price increase by 1.45 ¢/oz. relative to the surrounding area of Philadelphia (50). Bleich et al. reported that the relative price of taxed beverages increased by 1.81 ¢/oz. 1-year post-tax (69) and 2.06 ¢/oz. two-year post-tax (76) in Philadelphia compared to Baltimore, resulting in a tax passthrough rate of 120.4% (69) and 137% (76), respectively. Petimar et al. reported that taxed beverage prices increased by 1.02 ¢/oz., with 68% of the tax passed through to prices 2 years after tax implementation in Philadelphia compared to Baltimore (81). Cawley et al. reported that the price of taxed beverages increased by 0.83 ¢/oz. relative to untaxed beverages, and 55.3% of the tax was passed through to consumers at the Philadelphia International Airport (68). In Seattle, a 1.75 ¢/oz. soda tax raised prices of taxed beverages by 1.03 ¢/oz. relative to Portland, corresponding to a 59% tax passthrough rate (48). Jones-Smith et al. found that the soda tax was associated with a price increase of 1.58 ¢/oz. relative to its comparison area south of Seattle, and 90% of the tax was passed through to consumers (71).

**Table 3:** Estimated effects of a soda tax on price, consumption, purchases, or sales of beverages in the studies included in the review.

Study ID	Effects of a soda tax on consumption	Effects of a soda tax on sales or purchases	Effects of a soda tax on prices
1			1. For small-size beverages (≤ 33.8 oz), price increases in Berkeley relative to those in comparison cities were 0.69 ¢/oz. (95% CI = 0.36, 1.03) for soda, 0.47 ¢/oz. (95% CI = 0.08, 0.87) for fruit-flavored beverages, and 0.47 ¢/oz. (95% CI = 0.25, 0.69) for SSBs overall. A pass-through rate of 47% for SSBs overall. 2. For 2-liter bottles and multipacks of soda, relative price increases were 0.46 ¢/oz. (95% CI = 0.03, 0.89) and 0.49 ¢/oz. (95% CI = 0.21, 0.77). 3. No relative price increases for untaxed beverages overall.
2	1. Consumption of SSBs decreased by 21% in Berkeley and increased by 4% in comparison cities (*p* = 0.046), the ratio of post- to pre-tax consumption in Berkeley relative to comparison cities is 0.76, 95% CI = 0.58, 0.995. 2. Water consumption increased more in Berkeley (+63%) than in comparison cities (+19%; p < 0.01).		
3			1. Across all brands and sizes of products examined, 43.1% (95% CI = 27.7, 58.4%) of the Berkeley tax was passed on to consumers. 2. For each mile of distance between the store and the closest store selling untaxed SSBs, pass-through rose 33.3% for 2-liter bottles and 25.8% for 12-packs of 12-oz cans.
4	Households living in Alameda County increased their consumption of sugar-sweetened beverages by 8.89 oz. relative to other households in the U.S., increased their consumption of soda by 26.56 oz. relative to other households in the U.S., and by 37.15 oz. relative to residents of San Mateo, a neighboring county.		
5	Reductions in self-reported mean daily SSB intake in grams (−19.8%, *p* = 0.49) and mean *per capita* SSB caloric intake (−13.3%, *p* = 0.56) from baseline to post-tax were not statistically significant.	1. SSB sales in Berkeley stores declined 9.6% (p < 0.001, 95%CI = -9.9, −9.3%) compared to estimates if the tax were not in place, but rose 6.9% (*p* < 0.001, 95%CI = 6.3, 7.2) for non-Berkeley stores. 2. Sales of untaxed beverages in Berkeley stores rose by 3.5% (95%CI = 3.1, 3.9) versus 0.5% (95%CI = 0.1, 0.9) (both *p* < 0.001) for non-Berkeley stores.	1. Pass-through was complete in large chain supermarkets (+1.07¢/oz., p = 0.001) and small chain supermarkets and chain gas stations (1.31¢/oz., p = 0.004), partial in pharmacies (+0.45¢/oz., *p* = 0.03), and negative in independent corner stores and independent gas stations (−0.64¢/oz., *p* = 0.004). 2. Sales-unweighted mean price change from scanner data was +0.67¢/oz. (*p* < 0.001) (sales-weighted, +0.65¢/oz., *p* = 0.003, 95% CI = 0.23, 1.07), with +1.09¢/oz. (p < 0.001) for sodas and energy drinks, but a lower change in other categories. 3. One year following implementation of the nation’s first large SSB tax, prices of SSBs increased in many, but not all, settings.
6		Reduced supermarket purchases of soda in the taxing jurisdiction. Half of these reduced purchases are substituted to just outside the taxing jurisdiction.	Relative to an in-state synthetic control, per-ounce prices and volume-weighted per-ounce prices at the Berkeley supermarket are estimated to increase by a statistically significant 0.19 ¢ and 0.15 ¢, respectively. These constitute price increases of 2.95 and 4.35%, respectively, reflecting respective tax pass-through rates of 18.53 and 15.25%.
7			One month after tax implementation, prices had increased by 0.83 ¢/oz. (95% CI = 0.33, 1.33; p = 0.002) in taxed stores relative to untaxed stores, and 55.3% (95%CI = 22, 89%; *p* = 0.002) of the tax was passed on to consumers.
8			1. The tax was substantially, but not entirely, passed through to consumers in the form of higher prices; pass-through rates can vary across different localities. 2. The estimated pass-through based on posted prices at stores is 51.2%; whereas, pass-through based on register prices is 79.3%. 3. Data hand-collected from restaurants indicates that the pass-through of the tax was 69.4% on fountain drinks. 4. There is little evidence of any impact of the tax on the store prices of untaxed beverages. 5. The change of the taxed products retail register price relative to the prices in June in Boulder County and Fort Collins is 1.550, SE = 0.201.
9			Outside Philadelphia County, soda products prices increased, on average $0.0012/oz., while prices inside Philadelphia County prices increased, on average, $0.0165. The difference between these two increases is $0.0153. 100% excise tax pass-through rate on SSBs with almost no price change on substitute products.
10	Within the first 2 months of tax implementation, relative to the comparison cities, in Philadelphia the odds of daily consumption of regular soda was 40% lower (OR = 0.6, 95% CI = 0.37, 0.97); energy drink was 64% lower (OR = 0.36, 95% CI = 0.17, 0.76); bottled water was 58% higher (OR = 1.58, 95% CI = 1.13, 2.20); and the 30-day regular soda consumption frequency was 38% lower (OR = 0.62, 95% CI = 0.40, 0.98).		
11		1. There is a significant difference in the change of total beverage sales for those inside Philadelphia County versus those outside Philadelphia County (t = 5.35, p < 0.001). 2. Stores inside Philadelphia County experienced a significantly higher change (decrease in total beverage sales) in sales than those outsides of Philadelphia County (increase in total beverage sales). 3. This change did not coincide with a corresponding change in untaxed beverages.	
12		1. An increase in the beverage tax rate of 1 ¢/oz. decreases household purchases of taxed beverages by 53.0 oz. per month or 12.2%. 2. The decline concentrated in Philadelphia, where the tax decreased purchases by 27.7%, and there was no impact of the taxes in the other three cities combined.	
13	1. The estimated impact of a soda tax on the consumption of added sugars from SSBs and the frequency of consuming taxed beverages are negative but not statistically significant for children (a reduction of 2.4 g/day) and adults (a reduction of 5.9 g/day). 2. Due to soda tax, adults in Philadelphia consumed regular soda 10.4 fewer times per month, which is a reduction of approximately 30%.	The average amount of taxed beverages purchased per shopping trip decreased in Philadelphia and increased in comparison communities, which results in a relative decrease of 8.5 oz. per shopping trip at stores in Philadelphia.	
14	1. At baseline, SSBs were consumed 1.25 times/day (95% CI = 1.00, 1.50) in Berkeley and 1.27 times/day (95% CI = 1.13, 1.42) in comparison city. 2. Adjusting for covariates, consumption in Berkeley declined by 0.55 times/day (95% CI = -0.75, −0.35) for SSBs and increased by 1.02 times/day (95% CI = 0.54, 1.50) for water. 3. Changes in consumption in Berkeley were significantly different from those in the comparison group, which had no significant changes.		
15		1. In Philadelphia and Baltimore, taxed beverage volume sales in 4 weeks decreased in all store types. 2. Compared with Baltimore, there were significantly more substantial declines in the volume of taxed beverages sold in the after-tax period in Philadelphia. Total volume sales of taxed beverages in Philadelphia decreased by 1.3 billion oz. (from 2.475 billion to 1.214 billion) or by 51.0% following tax implementation.	1. In Philadelphia and Baltimore, the mean price per oz. of taxed beverages increased at all stores in the after-tax periods. 2. Compared with Baltimore, Philadelphia experienced significantly greater increases in taxed beverage prices. For supermarkets, the prices increased 0.65 ¢/oz., 95% CI = 0.60, 0.69; For mass merchandize stores, the price increased 0.87 ¢/oz., 95% CI = 0.72, 1.02; For pharmacies, the price increased 1.56 ¢/oz., 95% CI = 1.50, 1.62.
16		Soda sales fell significantly compared with control beverage groups in the period immediately following the election, decreasing by between 10 and 20% compared with precampaign levels. On-campus soda sales continued to fall when the tax was implemented in the city but not on campus—decreasing by 18–36% compared with the precampaign period—and remained at this depressed level after the tax implementation on campus.	
17		The volume sold of taxed beverages declined by 38.9% in Philadelphia after soda tax implementation compared to Baltimore.	The price increase of taxed beverages in Philadelphia was 1.81 ¢/oz. (95% CI = 1.52, 2.09) compared to Baltimore, revealing a 120.4% tax pass-through rate.
18	No evidence showed that substantial changes in the overall consumption of SSBs or added sugars consumed through beverages for either adults or children after the tax.	There was a slight decrease in the volume of SSBs purchased per shopping trip in Oakland and a small increase in purchases at stores outside of the city, resulting in a decrease in purchases of 11.33 oz., but it was not statistically significant.	Roughly 60% of the tax passed on to consumers in the form of higher prices.
19			1. The tax was fully passed through to consumers via higher retail prices. 2. For all taxed beverages combined, the 1.5 ¢-per-oz tax raised prices by 1.582 ¢/oz. (95% CI = 1.21, 1.89). 3. The impact of the tax is substantial, raising prices per oz. by 21% on average. Pass-through is complete for specific categories of taxed beverages, such as regular soda (1.591 ¢/oz), diet soda (1.551 ¢/oz), energy drinks (1.998 ¢/oz), and juice drinks (1.928 ¢/oz).
20			1. The average price of SSBs increased by 0.92 ¢/oz. (95% CI = 0.28, 1.56) in Oakland and 1.00 ¢/oz. (95% CI = 0.35, 1.65) in San Francisco, compared to prices in untaxed cities. 2. The soda tax did not significantly alter prices of water, 100% juice, or milk of any size examined. 3. Diet soda exhibited a higher price increase in taxed cities.
21			1. The soda tax was associated with an average price increase of 1.58 ¢/oz. among Seattle retailers. Nearly the full cost of the tax was passed through to consumers in Seattle. 2. Prices of some non-taxed beverages also increased while the prices of healthy foods generally did not.
22		The soda tax was associated with reductions of taxed beverage purchases at 3 and 6 month but not 12 month. Analyses aggregating all 6 weeks of post-tax time points showed significant reductions (−203.7 oz., 95% CI = -399.6, −7.8).	
23			1. There was a 82% tax pass-through for bottled regular soda one year after tax implementation, with a price increase of 8%. 2. No statistically significant change in prices was found in either time period for taxed and untaxed fountain drinks and untaxed bottled diet soda.
24		Volume sold of taxed beverages in Cook County compared with St Louis exhibited a posttax implementation level decrease of 25.7% (β = −0.297; 95%CI = −0.415, −0.179) and a posttax repeal level increase of 30.5%(β = 0.266, 95% CI = 0.124, 0.408), with no net change in volume sold from pretax to 8 months after repeal.	Compared with St Louis, posttax implementation in Cook County resulted in a level increase in taxed beverage prices of 1.13 ¢/oz. (95% CI = 1.01, 1.25), followed by a posttax repeal level decrease of −1.19 ¢/oz. (95%CI = −1.33, −1.04), with no resulting change pretax to posttax repeal.
25		1. Volume sold of taxed beverages decreased by 27% (95% CI = -30, −25%) in Cook County relative to St. Louis. 2. The magnitude of decrease in volume sold across types of taxed beverages was heterogeneous: −32% (95%CI = -35, −28%) for soda versus −11% (95%CI = -18, −3%) for energy drinks, −37% (95%CI = -41, −34%) for diet beverages versus −25% (95%CI = -28, −21%) for SSBs, and − 29% (95%CI = -32, −26%) for family-size versus −19% (95%CI = -21, −16%) for individual-size beverages. 3. There was no significant change in volume sold of untaxed beverages in Cook County and its border area.	
26		1. Volume sold of taxed beverage fell, on average, by 22% (*p* < 0.001) in the first year following the implementation of a soda tax. 2. Volume sold of taxed beverages fell to a greater extent for family-versus individual-size beverages (31% vs. 10%) and fell to a greater extent for soda (29%) compared to all other beverage types. 3. Moderate substitution to untaxed beverages was found—volume sold of untaxed beverages increased by 4% (*p* < 0.05). 4. There was no significant increase in the overall volume sold of taxed beverages in the 2-mile border area of Seattle.	On average, in the first year of post-tax implementation, prices of taxed beverages rose by 1.03 ¢/oz. (p < 0.001), corresponding to a 59% tax pass-through rate.
27			1. There was a 119% tax pass-through rate across all taxed beverages in Cook County compared to its comparison site. 2. This price increase was 34% for taxed beverages. 3. For untaxed beverages, prices increased slightly by 0.04 ¢/oz. driven mainly by an increase in milk prices (0.12 ¢/oz). 4. Pass-through was higher for individual-size (126%) compared to family-size (117%) beverages and higher for energy drinks (145%) compared to other sweetened beverages. 5. Based on the baseline prices of different categories and sizes of beverages, the effective percentage increase in beverage prices ranged from a 52% increase for family-size soda to a 10% increase for family-size energy drinks.
28	People in Philadelphia were more likely to decrease their frequency of SSB consumption (39.2% vs. 33.5%), and less likely to increase their frequency of SSB consumption (38.9% vs. 43.0%) relative to those residing in untaxed cities.		
29		1. Purchases of taxed beverages declined by 6.1 fl oz. (95% CI = −9.9, −2.4; p < 0.001), corresponding to a 42% decline in Philadelphia compared with Baltimore; there were no significant changes in purchases of nontaxed beverages. 2. Although there was no significant moderation by neighborhood income or customer education level, declines in taxed beverage purchases were larger among customers shopping in low-income neighborhoods (−7.1 fl oz.; 95%CI = −13.0, −1.1; p = 0.001) and individuals with lower education levels (−6.9 fl oz.; 95% CI = −12.5, −1.3; *p* = 0.001).	Taxed beverage prices increased 2.06 ¢/oz. (95% CI = 1.75, 2.38; p < 0.001), with 137% of the tax passed through to prices 2 years after tax implementation in Philadelphia compared to Baltimore
30			The tax was largely, but not completely, passed through to consumers. In both the hand-collected store data and restaurant data, pass-through is slightly less than 75%, whereas pass-through is just over 50% using the scanner data; consumers bear most, but not all, of the largest tax on sugar-sweetened beverages.
31	1. Intake of soda reduced by 0.81 servings per week in Philadelphia compared with all other comparison cities 2 years after tax implementation. 2. There was no significant difference in 100% juice or milk intake. 3. In subgroup analyses, the tax was associated with a reduction of 1.13 servings per week in Hispanic/Latinx adolescents (95%CI = −2.04, −0.23 servings; *p* = 0.01) and 1.2 servings per week in adolescents with obesity (95% CI = −2.33, −0.13 servings; p = 0.03).		
32		Taxed beverages sales fell by 14%, but 46% of this decrease is offset with an increase in the border area.	The taxed beverage prices increased by 0.49 ¢/oz. in Oakland relative to Sacramento. Tax pass-through is 49%.
33			Taxed beverage prices increased by 0.73 ¢/oz. (95% CI = 0.47,1.00) on average in supermarkets and grocery stores in Oakland relative to Sacramento and 0.74 ¢/oz. (95% CI = 0.39,1.09) in pharmacies, but did not change in convenience stores (−0.09 ¢/oz., 95% CI = −0.56,0.39). Untaxed beverage prices overall increased by 0.40 ¢/oz. (95% CI = 0.05,0.75) in pharmacies but did not change in other store types. Prices of taxed individual-size soda specifically increased in all store types, by 0.91–2.39 ¢/oz. (p < 0.05), as did prices of untaxed individual-size soda in convenience stores (0.79 ¢/oz., 95% CI = 0.01,1.56) and pharmacies (1.66 ¢/oz., 95% CI = 0.09,3.23).
34			1. In fast-food restaurants, the price of bottled regular soda increased by 1.44 ¢/oz. (95%CI = 0.50, 2.73), with tax passthrough rate of 144%. 2. The price of bottled diet soda increased by 1.17 cents/oz. (95%CI = 0.07, 2.13). 3. There were not statistically significant price effects for unsweetened beverages and fountain drinks.
35		1. Taxed beverages sales fell by 22% in Seattle relative to Portland. 2. Declines were larger for familysize (29%) compared to individual-size (10%) beverages; particularly for soda (36% decrease for family-size compared to no change for individual-size). 3. There was no change in volume sold of taxed beverages in Seattle’s 2-mile border area, suggesting no cross-border shopping.	Prices of taxed beverages increased by 1.04 ¢/oz. 2-year post-tax, with 59% tax pass-through rate.
36		A 5% volume reduction in Washington but fail to detect an effect in Berkeley.	Prices in Washington reacted sharply and promptly (often by a larger magnitude than the tax), whereas retail prices in Berkeley reacted marginally (by less than 30% the magnitude of the tax).
37		1. The total volume of taxed beverages per store sold in Philadelphia decreased by 46%. 2. A large amount of cross-shopping to stores outside of Philadelphia off-sets more than half of the reduction in sales in the city and reduces the net decrease in sales of taxed beverages to only 22%.	The tax is passed through at an average rate of 97%, leading to a 34% price increase.
38		Each cent per ounce of taxes causes the sales quantity of taxed beverages to decrease in a range of 5.1–14.4%.	Each cent per ounce of taxes causes the price of the taxed beverages to increase in a range from 0.47 to 0.98 ¢/oz.
39		1. Taxed beverage volume sold decreased by 18% in Oakland relative to Sacramento, with a larger decrease for family-size beverages (23%) relative to individual-size beverages (8%). 2. There was a 9% increase in volume sold of taxed beverages in the two-mile border area surrounding Oakland relative to the Sacramento border area, driven by a 12% increase for family-size taxed beverages. 3. After accounting for this cross-border shopping, there was a net decrease of 6% in taxed beverage volume sold in Oakland. 4. There was no significant change in untaxed beverage volume sold in either Oakland or its border area relative to their respective comparison sites.	Taxed beverage prices increased by 0.67 ¢/oz. in Oakland relative to Sacramento, corresponding to 67% pass-through.
40		1. Taxed beverage volume sales in stores decreased by 50% (95% CI = 36, 61%), volume sales of nontaxed beverages did not change after tax implementation. 2. After accounting for cross-border shopping, taxed beverage volume sales decreased in Philadelphia by 35% in 2018. Volume sales of nontaxed beverage concentrates increased on average by 34%, but there was no evidence of substitution to high-calorie foods.	After tax implementation, taxed beverage prices in Philadelphia increased by 1.02 ¢/oz. (95% CI = 0.94, 1.11; 68% pass through).

Second, soda taxes reduced SSB sales and purchases in the taxing jurisdictions relative to the nontaxing jurisdictions. SSB sales in Berkeley declined by 2.7% relative to neighboring cities (51). The total sales volume of taxed beverages decreased by 48.7% (49), or 38.9% (69) in Philadelphia relative to Baltimore following soda tax implementation. Similarly, the volume sold of taxed beverages decreased by 27% (74) or 25.7% (73) in Cook County relative to St. Louis. Sales volume of taxed SSBs reduced by 14% (77) or 18% (80) in Oakland compared to Sacramento. Taxed beverage volume sales decreased by 50% in Philadelphia relative to Baltimore two years post-tax (81). Taxed beverage sales fell by 22% in Seattle relative to Portland (79). Compared with the surrounding metropolitan area, sales quantity of taxed SSB fell by 10.6% in Berkeley, 14.4% in Oakland, 5.1% in Boulder, 8.3% in Philadelphia, 10.7% in Cook County, and 5.6% in Seattle, respectively (63). Concerning purchases, taxed beverage purchases declined by 6.1 fl oz., corresponding to a 42% decline in Philadelphia relative to Baltimore (76). An increase in the soda tax rate by 1 ¢/oz. decreased monthly household purchases of taxed beverages by 53.0 oz. or 12.2% (41). The average quantity of taxed beverages purchased per shopping trip decreased in Philadelphia and increased in neighboring cities, which resulted in a relative decrease of 8.5 oz. of SSBs purchased per shopping trip in Philadelphia (40).

Third, soda taxes reduced the consumption of SSBs in taxing jurisdictions relative to nontaxing jurisdictions. Consumption of SSBs was found to decrease by 25% in Berkeley relative to neighboring cities (46). Soda intake was reduced by 0.81 servings per week in Philadelphia compared with all other comparison cities two years after tax implementation (60). The odds of daily consumption of regular soda and energy drinks were 40 and 64% lower, respectively, in Philadelphia relative to the comparison cities two-month post-tax (52). Adults in Philadelphia were found to consume regular soda 10.4 fewer times per month after soda tax implementation, denoting a reduction of approximately 30% (40). People in Philadelphia were more likely to reduce their frequency of SSB consumption, and their monthly SSB consumption declined by 51.65 oz. compared to those residing in neighboring cities (58).

Fourth, the impact of soda taxes on SSB sales and purchases was compromised by cross-border shopping. Roberto et al. estimated that approximately a quarter of the decrease in the taxed beverage sales in Philadelphia could be offset by increases in sales in bordering areas (49). Seiler et al. estimated that cross-shopping to stores outside Philadelphia offset over half of the reduction in SSB sales in the city (50). Petimar et al. estimated that 30% of the reduction in taxed beverage sales in Philadelphia was offset by cross-border shopping (81). Leider et al. estimated that two-thirds of the overall decrease in the volume sold of taxed beverages in Oakland was offset by cross-border shopping (80). Léger and Powell estimated that 46% of Oakland’s taxed beverage sales decrease was offset by cross-border shopping (77). Powell and Leider reported that there was no significant change in taxed beverage sales in Seattle’s two-mile border area, suggesting there was no cross-border shopping for taxed beverages (79).

### 3.4. Meta-analysis

Table 4 summarizes the results from meta-analyses. Data from 27 studies contributed to the meta-analysis: 22 focused on the change in prices of taxed beverages and tax passthrough rate (37–39, 42, 45, 48–51, 68–73, 75–81), 11 on purchases of taxed beverages (41, 42, 48, 49, 69, 73, 74, 77, 79–81), three on the change in consumption of taxed beverages (46, 47, 52), 15 on the change in prices of untaxed beverages (38, 39, 42, 45, 49–51, 68, 71, 73, 75–78, 81), and five on the change in the purchases of untaxed beverages (48, 74, 77, 80, 81). Soda tax implementation was found to be associated with an increase in the prices of taxed beverages by 1.06 ¢/oz. (95% CI = 0.90, 1.22; *I*^2^ = 98.8%; RE), a reduction in the purchases of taxed beverages by 27.3% (95% CI = 19.3, 35.4%; *I*^2^ = 97.2%; RE). The soda tax passthrough rate was estimated to be 79.7% (95% CI = 65.8, 93.6%; *I*^2^ = 99.1%; RE). Following the soda tax implementation, the prices of untaxed beverages were estimated to increase by 0.08 ¢/oz. (95% CI = 0.04, 0.12; *I*^2^ = 77.3%; RE). No changes were observed for the purchases of untaxed beverages and the consumption of taxed beverages (*p* > 0.05). Meta-regression found that 1 ¢/oz. increase in the soda tax rate was associated with an increase in the prices of taxed beverages by 0.84 ¢/oz. (95% CI = 0.33, 1.35). No dose–response effect of soda taxes was found for purchases and consumption of taxed beverages or the tax passthrough rate (*ps* > 0.05). No publication bias was identified by Egger’s or Begg’s tests (*ps* > 0.05).

**Table 4:** Results from meta-analyses and publication bias tests.

Outcome	Studies included in the meta-analysis	*I*^2^ index	Pooled Effect size (95% CI)	Model	Publication bias test	
					*p*-value Egger’s test	*p*-value Begg’s test
Price change in taxed beverages (¢/oz)	Falbe et al. (45), Cawley and Frisvold (37), Silver et al. (51), Cawley et al. (38, 39), Roberto et al. (49), Bleich et al. (69), Cawley et al. (42), Falbe et al. (70), Jones-Smith et al. (71), Marinello et al. (72), Powell and Leider (73), Powell and Leider (48), Powell et al. (75), Bleich et al. (76), Léger and Powell (77), Marinello et al. (78), Powell and Leider (79), Seiler et al. (50), Leider and Powell (80), Petimar et al. (81)	98.8%	1.06 (0.90, 1.22)	RE	0.75	0.76
Price change in untaxed beverages (¢/oz)	Falbe et al. (45), Silver et al. (51), Cawley et al. (38, 39), Roberto et al. (49), Seiler et al. (50), Cawley et al. (42), Jones-Smith et al. (71), Powell and Leider (73), Powell et al. (75), Bleich et al. (76), Léger and Powell (77), Marinello et al. (78), Petimar et al. (81)	77.3%	0.08 (0.04, 0.12)	RE	0.08	0.55
Purchase change in taxed beverages	Cawley et al. (41), Roberto et al. (49), Cawley et al. (42), Powell and Leider (73), Powell and Leider (48), Powell et al. (74), Bleich et al. (69), Léger and Powell (77), Powell et al. (53, 54), Leider and Powell (80), Petimar et al. (81)	97.2%	−27.3% (−35.4, −19.3%)	RE	0.98	0.39
Purchase change in untaxed beverages	Powell et al. (74), Powell and Leider (48), Léger and Powell (77), Leider and Powell (80), Petimar et al. (81)	0.0%	3.1% (0.3, 5.9%)	FE	0.09	0.09
Consumption change in taxed beverages	Falbe et al. (46), Zhong et al. (52), Lee et al. (47)	48.7%	−36.1% (−51.4, −20.8%)	FE	0.59	1.00
SSB tax pass-through rate	Falbe et al. (45), Cawley and Frisvold (37), Silver et al. (51), Cawley et al. (38, 39), Roberto et al. (49), Bleich et al. (69), Cawley et al. (42), Falbe et al. (70), Jones-Smith et al. (71), Marinello et al. (72), Powell and Leider (73), Powell and Leider (48), Powell et al. (75), Bleich et al. (76), Léger and Powell (77), Marinello et al. (78), Powell et al. (53, 54), Seiler et al. (50), Leider and Powell (80), Petimar et al. (81)	99.1%	79.7% (65.8, 93.6%)	RE	0.44	0.22

RE: random-effect model, FE: fixed-effect model.

### 3.5. Study quality assessment

Table 5 reports criterion-specific and overall ratings from the study quality assessment. The included studies, on average, scored six out of ten, with scores ranging from 3 to 9. All studies collected and analyzed both pre- and post-tax outcomes for the intervention group (i.e., retailers or consumers residing in the taxing jurisdiction) and included a control group (i.e., retailers or consumers living outside the taxing jurisdiction or beverages not subject to the soda tax). The majority of studies (*n* = 36) had comparable intervention and control groups. Additionally, most studies had a sample representative of the stores or the population residing in the taxing jurisdiction (*n* = 17) (35, 36, 38–42, 44, 45, 50, 51, 60, 70–72, 74, 75). Furthermore, most studies included the change in beverage prices (*n* = 28) (36–39, 42, 43, 45, 48–51, 59, 61–63, 68–73, 75–81), sales or purchases (*n* = 22) (35, 36, 40–42, 44, 48–51, 56, 57, 62, 63, 69, 73, 74, 76, 77, 79–81) and consumption (*n* = 11) as outcomes (36, 40, 42, 44, 46, 47, 50–52, 58, 60). Additionally, several studies assessed the impact of cross-border sales or purchases (*n* = 17) (36, 37, 39, 40, 42, 46, 48–50, 57, 62, 69, 74, 77, 79–81). However, we noted some limitations in the quality of the included studies. Only two studies provided a sample size justification (58, 62), and nine studies performed adequate statistical procedures to adjust for temporal trends of the outcomes (37, 44, 47, 49, 50, 52, 60, 73, 79). To enhance the rigor of future research in this area, we recommend that studies provide clear justifications for sample sizes and utilize appropriate statistical procedures to account for temporal trends. Additionally, researchers should aim to improve reporting practices, including the transparent reporting of study limitations and potential sources of bias.

**Table 5:** Study quality assessment.

Study ID Criterion	1	2	3	4	5	6	7	8	9	10	11	12	13	14	15	16	17	18	19	20
1. Did the study collect and analyze both pre- and post-tax outcomes for the intervention group (i.e., retailers or consumers residing in the taxing jurisdiction)?	1	1	1	1	1	1	1	1	1	1	1	1	1	1	1	1	1	1	1	1
2. Did the study include a control group (i.e., retailers or consumers residing outside of the taxing jurisdiction, or beverages not subject to soda taxes)?	1	1	1	1	1	1	1	1	1	1	1	1	1	1	1	1	1	1	1	1
3. Were the intervention and control groups similar in all aspects except for their soda tax implementation status? If not, were adequate statistical procedures performed to adjust for group differences?	1	1	1	1	1	1	1	1	1	1	1	1	1	1	1	0	1	1	1	1
4. Were adequate statistical procedures performed to adjust for the temporal trends of the outcomes?	0	0	1	1	0	0	0	0	0	1	0	0	0	1	1	0	0	0	0	0
5. Was a sample size justification provided?	0	0	0	0	0	0	0	0	0	0	0	0	0	0	0	0	0	0	0	0
6. Was the study sample representative of the stores or the population residing in the taxing jurisdiction?	1	0	0	1	1	1	0	0	0	0	1	1	1	0	0	0	0	1	1	1
7. Was the change in beverage prices following the soda tax implementation included as an outcome?	1	0	1	0	1	1	1	1	1	0	0	0	0	0	1	0	1	1	1	1
8. Was the change in beverage sales or purchases following the soda tax implementation included an outcome?	0	0	0	1	1	1	0	0	0	0	1	1	1	0	1	1	1	1	0	0
9. Was the change in beverage consumption following the soda tax implementation included as an outcome?	0	1	0	1	1	1	0	0	0	1	0	0	1	1	0	0	0	1	0	0
10. Did the study assess the impact of cross-border sales or purchases following the soda tax implementation?	0	1	1	0	0	1	0	0	0	0	0	0	1	0	1	0	1	1	1	0
Total score	5	5	6	7	7	8	4	4	4	5	5	5	7	5	7	3	6	8	6	5

Study ID Criterion	21	22	23	24	25	26	27	28	29	30	31	32	33	34	35	36	37	38	39	40
1. Did the study collect and analyze both pre- and post-tax outcomes for the intervention group (i.e., retailers or consumers residing in the taxing jurisdiction)?	1	1	1	1	1	1	1	1	1	1	1	1	1	1	1	1	1	1	1	1
2. Did the study include a control group (i.e., retailers or consumers residing outside of the taxing jurisdiction, or beverages not subject to soda taxes)?	1	1	1	1	1	1	1	1	1	1	1	1	1	1	1	1	1	1	1	1
3. Were the intervention and control groups similar in all aspects except for their soda tax implementation status? If not, were adequate statistical procedures performed to adjust for group differences?	1	1	1	1	0	1	1	0	1	1	0	1	1	0	1	1	1	1	1	1
4. Were adequate statistical procedures performed to adjust for the temporal trends of the outcomes?	0	0	0	1	0	0	0	0	0	0	1	0	0	0	1	0	1	0	0	0
5. Was a sample size justification provided?	0	0	0	0	0	0	0	1	0	0	0	0	0	0	0	1	0	0	0	0
6. Was the study sample representative of the stores or the population residing in the taxing jurisdiction?	1	0	1	0	1	0	1	0	0	0	1	0	0	0	0	0	1	0	0	0
7. Was the change in beverage prices following the soda tax implementation included as an outcome?	1	0	1	1	0	1	1	0	1	1	0	1	1	1	1	1	1	1	1	1
8. Was the change in beverage sales or purchases following the soda tax implementation included an outcome?	0	1	0	1	1	1	0	0	1	0	0	1	0	0	1	1	1	1	1	1
9. Was the change in beverage consumption following the soda tax implementation included as an outcome?	0	0	0	0	0	0	0	1	0	0	1	0	0	0	0	0	1	0	0	0
10. Did the study assess the impact of cross-border sales or purchases following the soda tax implementation?	0	1	0	0	1	1	0	0	0	0	0	1	0	0	1	1	1	0	1	1
Total score	5	5	5	6	5	6	5	4	5	4	5	6	4	3	7	7	9	5	6	6

For each criterion, a score of one was assigned if “yes” was the response, whereas a score of zero was assigned otherwise. A study-specific global score ranging from 0 to 10 was calculated by summing up scores across all criteria. The study quality assessment helped measure the strength of scientific evidence but was not used to determine the inclusion of studies.

## 4. Discussion

A few previous meta-analyses estimated the effect of soda taxes or SSB prices on beverage sales, purchases, and consumption. Nakhimovsky et al. reviewed studies in middle-income countries and estimated that a 10% increase in SSB prices was associated with a reduction in SSB consumption from 1.2–9.3 kcal per person per day (83). Teng et al. estimated that a 10% soda tax was associated with a decline in SSB purchases and consumption by 10% (84). Escobar et al. (14) and Powell et al. (85) estimated the price-elasticity of SSBs to be −1.30 and − 1.21, indicating that a 10% increase in SSB prices was associated with a reduction in demand for SSBs by 13.0 and 12.1%, respectively. Afshin et al. (12) reported that a 10% increase in SSB prices was associated with a reduction in SSB consumption by 7%. Those estimates from the previous meta-analyses were comparable to the estimated impact of soda taxes on SSB purchases and consumption in this review. However, the key differences are: previous reviews and meta-analyses were nearly exclusively based on the “proxy” or “modeling” studies due to lacking a soda tax in reality and collected data from multiple other countries besides the US. This review contributed to the literature by comprehensively assessing the efficacy of soda taxes based on evidence reported exclusively from natural experiments (the “local” studies). In addition, the review provided quantitative estimates of the magnitude of the tax effect. Review findings can potentially inform policymakers in designing and implementing soda taxes to curb the obesity epidemic.

Several main criticisms of soda taxes are worth noting. First, substitution effects in addition to cross-border shopping may partially offset the intended effect of soda taxes on SSB purchases and consumption (49, 50). A nationwide soda tax adoption could eliminate cross-border shopping (49). Second, the soda tax could be regressive and the impact of soda consumption is greatest in low income populations. However, a systematic review revealed only modest differences—0.1–1.0% and 0.03–0.60% of annual household income paid for soda taxes for low- and high-income households, respectively (86). Third, revenues collected by soda taxes were rarely earmarked for healthy diet promotion, such as in the form of healthy food subsidies paid to low-income households (87). Fourth, relatively arbitrary classifications of SSBs result in taxing some low-sugar beverages but exempting some high-sugar ones (88). The beverage industry has used these policy loopholes to lobby against soda tax bills (88). Finally, some researchers questioned the efficiency of the volumetric soda tax and suggested taxing the amount of sugar in a drink rather than the volume of liquid that accompanied the sugar to boost the soda tax’s health benefits and overall economic gains (89).

Despite the various criticisms of soda taxes, it remains a potentially effective policy leverage to nudge people toward reducing purchases and consumption of SSBs. There is also preliminary evidence linking soda tax to reduced body weight, BMI, and overweight or obesity (14, 15). The revenues collected from soda taxes could support other health interventions, such as nutrition education campaigns or healthy food subsidies, which may reinforce the long-term sustainability of soda taxes on dietary pattern changes. Such policy arrangements might also alleviate health disparities at the population level.

This study comprehensively reviews evidence reported from natural experiments on soda taxes implemented across US local taxing jurisdictions. The strengths of the included studies encompassed real-world policy interventions providing causal inferences, the inclusion of comparison cities, an elucidation of the local soda tax implementation processes, and longitudinal changes in price, consumption, and purchases pre-and post-tax. However, a few limitations of this review and the included studies should be noted. First, all studies were natural experiments focused on one or a few US cities implementing a soda tax. The limited geographic coverage and nationally non-representative sample have confined the generalizability of study findings. Therefore, caution should be exercised when extrapolating the results to other cities or jurisdictions. Second, DID is a quantitative method to reveal causal references, but the resulting evidence should not be overstated due to lacking a randomization design (82). Third, it is important to consider the magnitude of the tax rates examined in the included studies. The range of tax rates observed in the reviewed studies was relatively narrow, typically between 1 and 2 ¢/oz. Thus, the effects reported in the included studies may not necessarily apply to different tax magnitudes. Non-linearities in the effects on tax pass-through or purchases may exist, and these non-linearities could be influenced by factors such as company profit considerations or consumer price sensitivity. Additionally, we acknowledge that the slight increase in prices of untaxed beverages following soda tax implementations is an interesting finding. However, the reasons behind this increase are not entirely clear from available evidence. It could be a result of temporal changes in the prices of untaxed beverages or it may reflect substitution effects, where higher demand for untaxed beverages leads to a price increase. Further research is needed to understand the underlying factors contributing to this observation.

## 5. Conclusion

This study systematically reviewed evidence from natural experiments regarding the impact of soda taxes on beverage prices, sales, purchases, and consumption in the US. Soda tax implementation was associated with increased prices of taxed beverages and reduced purchases and consumption of taxed beverages. Soda taxes could be effective policy leverage to nudge people toward purchasing and consuming fewer SSBs. Future research should examine evidence-based classifications of SSBs, more targeted use of revenues generated by taxes to reduce health and income disparities, and the feasibility of redesigning the soda tax to improve its efficiency.

## Data availability statement

The original contributions presented in the study are included in the article/Supplementary material, further inquiries can be directed to the corresponding author.

## Author contributions

JS, JW, and RA designed the study, wrote the manuscript, and revised the manuscript. JW, FY, and JS jointly designed the search algorithm and screened articles. JS and JW performed data extraction and constructed the summary tables. All authors contributed to the article and approved the submitted version.

## Funding

This research was funded by the Fundamental Research Funds for the Central Universities, CUGB, grant number 2-9-2020-036; The Ministry of Education of Humanities and Social Science project, grant number 19YJC890044.

## Conflict of interest

The authors declare that the research was conducted in the absence of any commercial or financial relationships that could be construed as a potential conflict of interest.

## Publisher’s note

All claims expressed in this article are solely those of the authors and do not necessarily represent those of their affiliated organizations, or those of the publisher, the editors and the reviewers. Any product that may be evaluated in this article, or claim that may be made by its manufacturer, is not guaranteed or endorsed by the publisher.
