# Supplementary material for: Impact of soda tax on beverage price, sale, purchase, and consumption in the US: a systematic review and meta-analysis of natural experiments
Source: Front Public Health. 2023 Sep 22;11:1126569. doi: 10.3389/fpubh.2023.1126569 (PMC10556476; doi:10.3389/fpubh.2023.1126569)
Supplement: Supplementary file 1 [file Table_1.DOCX]

**Appendix 1** Search algorithm in PubMed

(“taxes”[MeSH] OR “taxes” OR “tax” OR “taxation” OR “taxing” OR “taxed” OR “excise” OR “excises” OR “duty” OR “duties” OR “levy” OR “levies” OR “tariff” OR “tariffs” OR “pre-taxation” OR “post-taxation” OR “pre-tax” OR “post-tax”) AND (“beverages”[MeSH] OR “beverages” OR “beverage” OR “drink” OR “drinks” OR “drinking” OR “juice” OR “juices” OR “coffee” OR “milk” OR “tea” OR “water” OR “soda” OR “sodas” OR “cola” OR “colas” OR “coke” OR “softdrink” OR “softdrinks” OR “fruit punch” OR “lemonade” OR “carbonated” OR “sugar-sweetened-beverages” OR “SSB” OR “SSBs”) AND (“United States”[MeSH] OR “United States” OR “U.S.” OR “America” OR “American” OR “Americans” OR “USA”) AND (English [lang])
